# Editorial: AI for design and control of advanced robots

**DOI:** 10.3389/frobt.2026.1850311

**Published:** 2026-04-28

**Authors:** Guimin Chen, Xiaohu Li, Ke Wu, Peng Xia

**Affiliations:** 1 Xi’an Jiaotong University, Xi’an, China; 2 Mohamed bin Zayed University of Artificial Intelligence (MBZUAI), Abu Dhabi, United Arab Emirates; 3 The First Affiliated Hospital of Xi’an Jiaotong University, Xi’an, China

**Keywords:** artificial intelligence, decision making, digital twin, robotic control, robotic design

Artificial intelligence, machine learning, and data-driven methods have profoundly reshaped the design, modeling, interaction, and intelligent control of advanced robotic systems. Robots are evolving from single-function automated devices to intelligent agents capable of operating in unstructured, dynamic, and human-centered environments. Driven by this trend, the Research Topic “AI for Design and Control of Advanced Robots” in *Frontiers in Robotics and AI* aims to gather Frontier research that integrates AI methodologies with robotic design and control, addressing key challenges in system design, human–robot interaction, medical robotics, legged locomotion, and cognitive architectures for real-time autonomy.

This Research Topic presents four high-quality contributions covering original research, mini-review, and hypothesis-and-theory articles, spanning mixed-reality digital twin systems, medical robotic design and learning-based modeling, imitation learning for legged locomotion, and cognitive control structures inspired by global workspace theory. Together, they reflect the diversity, depth, and practical impact of AI-enabled robotics.

The hypothesis-and-theory article “Hypothesis on the functional advantages of the selection-broadcast cycle structure: global workspace theory and dealing with a real-time world,” by Nakanishi et al., explores a cognitive architecture inspired by global workspace theory. The authors hypothesize that a selection–broadcast cycle structure endows robotic systems with superior real-time decision-making, attention management, and adaptive behavior in dynamic environments. This theoretical work offers a biologically inspired perspective for building next-generation robots with higher-level cognitive and control capabilities.

The mini-review “Imitation learning for legged robot locomotion: a survey,” by Mirza and Singh, systematically reviews state-of-the-art imitation learning methods applied to legged robot locomotion. The article categorizes key approaches, discusses challenges such as sample efficiency, sim-to-real transfer, and robustness, and outlines future directions toward adaptive, versatile, and real-world deployable legged robots. This survey provides a valuable roadmap for researchers pursuing learning-based agile locomotion.

“Robotic transesophageal echocardiography: system design and deep learning-based kinematic modeling,” by Sajadi et al., addresses a critical need in minimally invasive medical robotics. The authors present a robotic system for transesophageal echocardiography (TEE) and develop a deep learning–based kinematic modeling approach to improve motion accuracy and safety. This work demonstrates how AI-driven modeling can enhance the precision, stability, and clinical reliability of medical robots, supporting safer and more standardized interventions in cardiac imaging and diagnosis.

Finally, “Design of a mixed reality–based digital twin human–machine interaction system for special working conditions,” by Li et al., proposes a digital twin human–machine interaction framework built on mixed reality (MR). Targeting hazardous, remote, or extreme working scenarios, the system integrates real-time perception, virtual–real fusion, and immersive operation to enhance safety, transparency, and operational efficiency. By combining digital twin modeling with MR-based interaction, the work provides a scalable solution for intuitive teleoperation and monitoring of robots in challenging environments, bridging the gap between physical robots and human operators.

Collectively, these four articles illustrate how AI innovations strengthen robotic design, interaction, modeling, locomotion, and cognitive control. They address real-world demands in special operations, healthcare, agile mobility, and real-time autonomy, while advancing both methodological and theoretical foundations of the field.

We sincerely thank all authors for their contributions, the reviewers for their rigorous and constructive feedback, and the editorial office of *Frontiers in Robotics and AI* for their strong support. We hope this Research Topic inspires further cross-disciplinary research and collaboration, accelerating the translation of AI-driven design and control into reliable, intelligent, and human-friendly robotic systems.

